# Roles of NF-κB in Cancer and Inflammatory Diseases and Their Therapeutic Approaches

**DOI:** 10.3390/cells5020015

**Published:** 2016-03-29

**Authors:** Mi Hee Park, Jin Tae Hong

**Affiliations:** College of Pharmacy and Medical Research Center, Chungbuk National University, 194-31, Osongsaengmyeong 1-ro, Osong-eup, Cheongwon-gun, Chungbuk 28160, Korea; pmh5205@hanmail.net

**Keywords:** NF-κB, canonical pathway, non-canonical pathway, cancer, inflammatory disease, therapeutic approaches

## Abstract

Nuclear factor-κB (NF-κB) is a transcription factor that plays a crucial role in various biological processes, including immune response, inflammation, cell growth and survival, and development. NF-κB is critical for human health, and aberrant NF-κB activation contributes to development of various autoimmune, inflammatory and malignant disorders including rheumatoid arthritis, atherosclerosis, inflammatory bowel diseases, multiple sclerosis and malignant tumors. Thus, inhibiting NF-κB signaling has potential therapeutic applications in cancer and inflammatory diseases.

## 1. Introduction

The nuclear factor-κB (NF-κB) family of transcription factors control the expression of genes involved in many critical physiological responses such as inflammatory responses, proliferation, differentiation, cell adhesion and apoptosis [1]. NF-κB transcription complexes have a variety of homo- and heterodimers consisting of the subunits p50, p52, c-Rel, RelA (p65) and RelB [2]. NF-κB signaling pathways can be divided into canonical and noncanonical pathways. In the canonical pathway, I kappa B kinase (IKK) phosphorylates IκBα at two N-terminal serines, triggering its ubiquitination and proteasomal degradation; this leads to the nuclear translocation of NF-κB complexes, predominantly p50/RelA and p50/c-Rel dimers [3]. The noncanonical NF-κB pathway involves different signaling molecules and leads to the activation of the p52/RelB dimer [4].

NF-κB is able to induce several of these cellular alterations and has been shown to be constitutively activated in some types of cancer cells. Constitutively activated NF-κB transcription factors have been associated with several aspects of tumorigenesis, including promoting cancer-cell proliferation, preventing apoptosis, and increasing a tumor's angiogenic and metastatic potential. Activation of the NF-κB/Rel by nuclear translocation plays a role in inflammation through induction of transcription of several proinflammatory genes [5]. Recent data indicate that activation of IKK-β, rather than IKK-α, participates in the primary pathway of proinflammatory genes [6]. IKK-β is expressed in fibroblast-like synoviocytes and plays a central role in TNF-α–mediated NF-κB activation and expression of proinflammatory genes [7]. IKK-β also activates NF-κB and inflammatory gene transcription in monocytes and CD4+ T lymphocytes [7]. Many natural products and drugs that have been involved in anti-cancer and anti-inflammatory activity have also been shown to inhibit NF-κB.

This review provides the signaling mechanisms and biological functions of the NF-κB pathway, and the role of NF-κB in cancer and inflammatory diseases, and the multitude of NF-κB inhibitors that have been reported.

## 2. NF-κB

Nuclear factor-κB (NF-κB) is a transcription factor that plays a crucial role in various biological processes, including immune response, inflammation, cell growth and survival, and development [1,8]. NF-κB is activated by various inflammatory stimuli such as growth factors and infectious microbes. NF-κB controls expression of a number of genes that regulate immune responses, cell growth and proliferation, survival and apoptosis, stress responses and embryogenesis and development of a variety of stimuli [7,9]. NF-κB is critical for human health, and aberrant NF-κB activation contributes to development of various autoimmune, inflammatory and malignant disorders including rheumatoid arthritis, atherosclerosis, inflammatory bowel diseases, multiple sclerosis and malignant tumors [10,11].

### 2.1. NF-κB Subunits

The mammalian NF-κB family is composed of five members, including RelA (p65), RelB, c-Rel, NF-κB1 p50, and NF-κB2 p52, which form various dimeric complexes that transactivate numerous target genes via binding to the κB enhancer [2]. The NF-κB proteins are normally sequestered in the cytoplasm by a family of inhibitors, including IκBα and other ankyrin repeat-containing proteins [6,12]. Proteasome-mediated processing of p105 and p100 produces the mature NF-κB1 and NF-κB2 proteins (p50 and p52) and results in disruption of the IκB-like function of these precursor proteins [13].

The NF-κB transcription factor family in mammals consists of five proteins including p65 (RelA), RelB, c-Rel, p105/p50 (NF-κB1), and p100/52 (NF-κB2) that associate with each other to form distinct transcriptionally active homo- and heterodimeric complexes [13,14]. Through combinatorial associations, the Rel protein family members can form up to 15 different dimers. Among them, the p50/65 heterodimer clearly represents the most abundant of Rel dimers, being found in almost all cell types [15]. In addition, dimeric complexes of p65/p65, p65/c-Rel, p65/p52, c-Rel/c-Rel, p52/c-Rel, p50/c-Rel, p50/p50, RelB/p50, and RelB/p52 have been described, some of them only in limited subsets of cells [10,11,12]. NF-κB family shares a Rel homology domain in their N-terminus. A subfamily of NF-κB proteins, including RelA (p65), RelB and c-Rel has a transactivation domain in their C-termini [16,17]. After processing of p105 and p100 by the ubiquitin/proteasome pathway that involves selective degradation of their C-terminal region containing ankyrin repeats, mature NF-κB subunits such as p50 and p52 are generated [16,17]. Actually, the p50 and p52 proteins have no intrinsic ability to activate transcription and act as transcriptional repressors when binding ΚB elements as homodimers [18].

### 2.2. NF-κB Signaling Pathway

The NF-κB dimers are sequestered in the cytoplasm by a family of inhibitors, called IκBs (Inhibitor of κB), which are proteins that contain multiple copies of a sequence called ankyrin repeats, in unstimulated cells [5,14]. The IκB proteins mask the nuclear localization signals (NLS) of NF-κB proteins and keep them sequestered in an inactive state in the cytoplasm by virtue of their ankyrin repeat domains [11,16]. Because the presence of ankyrin repeats in their C-terminal halves, p105 and p100 also function as IκB proteins. The C-terminal half of p100, that is often referred to as IκBδ, also functions as an inhibitor [19]. IκBδ degradation in response to developmental stimuli, such as those transduced through LTβR, potentiate NF-κB dimer activation in a NIK dependent non-canonical pathway [20].

#### 2.2.1. Canonical Pathway

**Figure 1:**
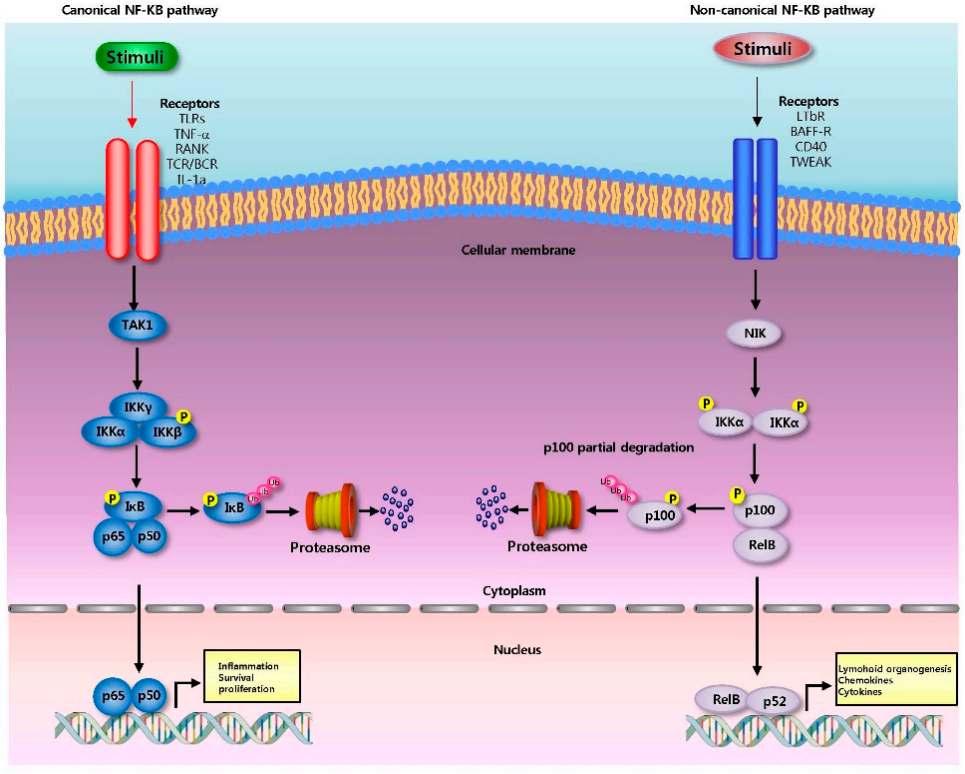
The canonical NF-κB pathway (**left**) induced by signals including antigens, TLR ligands and cytokines such as TNF uses a wide variety of signaling adaptors to engage and activate the IKKβ subunit of the IKK complex. IKKβ phosphorylation of IκB proteins bound to NF-κB dimers results in ubiquitination (Ub) of IκB and proteasome-induced degradation. This allows NF-κB to enter the nucleus and be involved in controlling the transcription of gene encoding functions as diverse as inflammation, cell survival and cell division. The noncanonical pathway (**right**) engaged in by members of the TNF-like family of cytokines requires NIK to activate IKKα, which then phosphorylates p100 (NF-κB2), triggering its proteosomal processing needed for the activation of p52-RelB dimers. Among its functions, this specific NF-κB heterodimer controls gene expression crucial for lymphoid organogenesis.

Canonical NF-κB pathway of NF-κB is activated after degradation of IκBα, which results in nuclear translocation of various NF-κB complexes, predominantly the p50/p65 dimer [3] (Figure 1). The degradation of IκBα is mediated by phosphorylation through the IκB kinase (IKK), a trimeric complex composed of two catalytic subunits, IKKα and IKKβ, and a regulatory subunit, IKKγ (also termed NF-κB essential modulator or NEMO) [21]. When activated by signals, the IκB kinase phosphorylates two serine residues located in an IκB regulatory domain [19,20]. When phosphorylated on these serines (e.g., serines 32 and 36 in human IκBα), the IκB inhibitor molecules are processed by ubiquitination, which then leads them to be degraded by a cell structure called the proteasome [22,23] . With the degradation of IκB, the NF-κB complex then enters into the nucleus where it can 'turn on' the expression of several genes that have DNA-binding sites for NF-κB [22,23]. The activation of these genes by NF-κB then leads to the given physiological response, for example, an inflammatory or immune response, a cell survival response, or cellular proliferation [24]. NF-κB turns on expression of its own repressor, IκBα. The newly synthesized IκBα then re-inhibits NF-κB and forms an auto feedback loop, which results in oscillating levels of NF-κB activity [22,23]. Genetic evidence suggests that this NF-κB pathway regulates important biological functions, such as lymphoid organogenesis, B-cell survival and maturation, dendritic cell activation, and bone metabolism.

#### 2.2.2. Non-Canonical Pathway

The non-canonical NF-κB pathway activates the RelB/p52 NF-κB complex using a mechanism that relies on the inducible processing of p100 instead of degradation of IκBα (Figure 1). The processing of p100 serves to both generate p52 and induce the nuclear translocation of the RelB/p52 heterodimer [4]. The discovery of non-canonical NF-κB signaling pathway came from the study of p100 processing [25]. In contrast to the constitutive and co-translational processing of p105, the processing of p100 is tightly regulated. In most cell types, p100 is the predominant product of NF-κB2 [26,27]. Overexpressed p100 is barely converted to p52 in mammalian cells, as opposed to the constitutive production of p50 from p105 [25]. However, p52 is actively generated in specific cell types, such as B cells, leading to the idea that p100 processing might be a signal-regulated event [25,28]. Indeed, the NF-κB-inducing kinase (NIK) induces p100 processing and is required for p100 processing in splenocytes. Moreover, endogenous p100 processing can be activated by various receptor signals in an NIK-dependent manner [29,30].

In this pathway, activation of the NF-κB inducing kinase (NIK) led to the phosphorylation and subsequent proteasomal processing of the NF-κB2 precursor protein p100 into mature p52 subunits in an IKK1/IKKa dependent manner [31]. Then, p52 dimerizes with RelB to appear as a nuclear RelB/p52 DNA binding activity and regulate a distinct class of genes [32]. In contrast to the canonical signaling that relies upon NF-kB essential modulator (NEMO)-IKK2 mediated degradation of IκBα, -β, -ε, the non-canonical signaling critically depends on NIK mediated processing of p100 into p52 [30,31]. Recent studies showed that synthesis of the constituents of the non-canonical pathway, RelB and p52, is controlled by the canonical IKK2-IκB-RelA: p50 signaling [30,31]. These studies suggest that an integrated NF-κB system network underlies activation of both RelA and RelB containing dimer and that a malfunctioning canonical pathway will lead to an aberrant cellular response also through the non-canonical pathway [30,31]. Deregulated non-canonical NF-κB signaling is associated with lymphoid malignancies [28,33]. Cell-differentiating or developmental stimuli such as B-Cell activation factor (BAFF), receptor activator of nuclear factor kappa-B ligand (RANKL) or lymphotoxin-α, activate the non-canonical NF-κB pathway [34].

## 3. Role of NF-κB in Diseases

**Figure 2:**
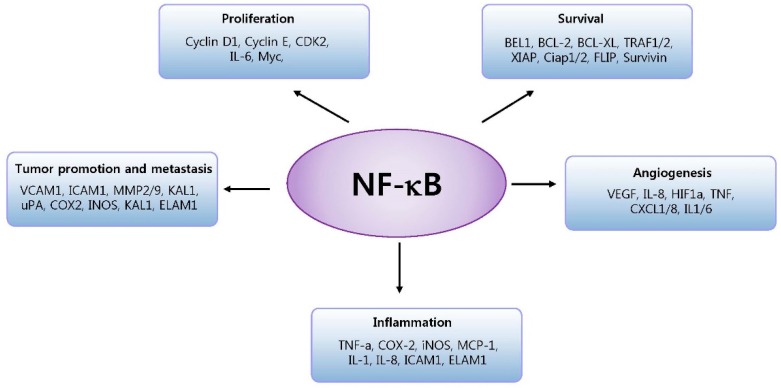
NF-κB activation affects hallmarks of cancer and inflammatory diseases through the transcription of genes involved in cell proliferation, survival, angiogenesis, inflammation and tumor promotion and metastasis. BCL2, B-cell lymphoma protein 2; BCL-XL, also known as BCL2-like 1; BFL1, also known as BCL2A1; CDK2, cyclin-dependent kinase 2; COX2, cyclooxygenase 2; CXCL, chemokine (C-X-C motif) ligand; DR, death receptor; ELAM1, endothelial adhesion molecule 1; FLIP, also known as CASP8; GADD45beta, growth arrest and DNA-damage-inducible protein beta; HIF1alpha, hypoxia-inducible factor 1alpha; ICAM1, intracellular adhesion molecule 1; IEX-1L, radiation-inducible immediate early gene (also known as IER3); IL, interleukin; iNOS, inducible nitric oxide synthase; KAL1, Kallmann syndrome 1 sequence; MCP1, monocyte chemoattractant protein 1 (also known as CCL2); MIP2, macrophage inflammatory protein 2; MMP, matrix metalloproteinase; MnSOD, manganese superoxide dismutase (also known as SOD2); TNF, tumour necrosis factor; TRAF, TNF receptor-associated factor; uPA, urokinase plasminogen activator; VCAM1, vascular cell adhesion molecule 1; VEGF, vascular endothelial growth factor; XIAP, X-linked inhibitor of apoptosis protein.

NF-κB activation affects hallmarks of cancer and inflammatory diseases through the transcription of genes involved in cell proliferation, survival, angiogenesis, inflammation and tumor promotion and metastasis as shown in Figure 2.

### 3.1. NF-κB and Cancer

NF-κB regulates the genes that control cell proliferation and cell survival [35]. Many different types of human tumors have misregulated NF-κB; that is, NF-κB is constitutively active. Active NF-κB turns on the expression of genes that keeps the cell proliferating and protects the cell from conditions that would otherwise cause it to die via apoptosis [36]. Cancer-associated chromosomal translocations, deletions and mutations might also disrupt genes that encode NF-κB and IκB proteins, uncoupling NF-κB factors from their regulators and causing constitutive NF-κB activation [37]. Constitutively activated NF-κB transcription factors have been associated with several aspects of tumorigenesis, including promoting cancer-cell proliferation, preventing apoptosis, and increasing a tumor's angiogenic and metastatic potential [37,38]. In tumor cells, NF-κB is consequently activated because mutations in genes encoding the NF-κB transcription factors themselves or in genes that control NF-κB activity. In addition, some tumor cells secrete factors that cause NF-κB to become active [39,40]. Blocking NF-κB can cause tumor cells to stop proliferating, to die, or to become more sensitive to the action of anti-tumor agents [41]. NF-κB stimulates the transcription of genes that encode G1 cyclins [1,40]. A κB site is present within the cyclin D1 promoter and there is strong evidence that NF-κB dependent cyclin D1 induction drives the proliferation of mammary epithelial cells during pregnancy [42,43]. NF-κB is also an inhibitor of programmed cell death [44,45]. This factor activates the transcription of several target genes that block the induction of apoptosis by TNF-α and other pro-apoptotic members of this family [22]. The anti-apoptotic factors that are induced by NF-κB include cellular inhibitors of apoptosis (cIAPs), caspase-8/FADD (FAS-associated death domain)-like IL-1beta-converting enzyme (FLICE) inhibitory protein (c-FLIP) and members of the BCL2 family (such as A1/BFL1 and BCL-XL) [22]. Cells with elevated NF-κB activity deregulate production of chemokines, which increases migratory activity [1]. At least one NF-κB-regulated chemokine, IL-8, has been shown to promote angiogenesis [46]. In addition, κB sites were identified in the promoters of genes that encode several matrix metalloproteinases (MMPs) that are proteolytic enzymes involved in promoting tumor invasion of surrounding tissue [47]. NF-κB contributes to extracellular matrix destruction by cancer cells [48,49]. NF-κB has also been shown to be involved in the development of carcinomas—cancers of epithelial origin, such as breast cancer [50]. Several studies have documented elevated or constitutive NF-κB DNA-binding activity both in mammary carcinoma cell lines and primary breast cancer cells [51,52].

In inflammatory cells, continuous NF-κB activity could promote the production of reactive oxygen species, thereby damaging DNA of surrounding epithelial cells [53]. Some of the best circumstantial evidence that supports such a role for NF-κB comes from various gastrointestinal cancers [54]. NF-κB activation is also associated with colorectal cancer. Colon cancer cell lines, human tumor samples, and stromal macrophages in sporadic adenomatous polyps also have increased NF-κB activity [55]. It has been shown that canonical NF-κB is a Fas transcription activator and the alternative NF-κB is a Fas transcription repressor [56]. 

### 3.2. NF-κB and Inflammatory Disease

NF-κB is a major transcription factor that regulates genes responsible for both the innate and adaptive immune response [57]. After activation of T- or B-cell receptors, NF-κB is activated through distinct signaling [58]. Upon ligation of the T-cell receptor, protein kinase Lck is recruited and phosphorylates the immunoreceptor tyrosine-based activation motifs (ITAMs) of the CD3 cytoplasmic tail [59]. ZAP70 is then recruited to the phosphorylated ITAMs and helps recruit Linker-for-activation of T cells (LAT) and Phospholipase C (PLC)-γ, which causes activation of Protein kinase C (PKC) [60]. Through a cascade of phosphorylation, the kinase complex is activated and NF-κB enter the nucleus to upregulate genes involved in T-cell proliferation, maturation and development [61]. NF-κB is chronically activated in many inflammatory diseases, including inflammatory bowel disease, arthritis, sepsis, gastritis, asthma, atherosclerosis and others [62]. It is important to note, though, that elevation of some NF-κB activators, such as osteoprotegerin (OPG), are associated with elevated mortality, especially from cardiovascular diseases [63]. Elevated NF-κB has also been associated with schizophrenia [64]. During inflammation, the function of a cell depends on signals in response to contact with adjacent cells and to combinations of hormones, especially cytokines that act on it through specific receptors [65]. A cell’s phenotype within a tissue develops through mutual stimulation of signals that coordinate its function with other cells, because cells alter their phenotype, and gradually express combinations of genes that prepare the tissue for regeneration after the cause of inflammation is removed [66]. Feedback responses that develop between tissue resident cells, and circulating cells of the immune system are important [66]. Fidelity of feedback responses between diverse cell types and the immune system depends on the integrity of mechanisms that limit the range of genes activated by NF-κB, allowing only expression of genes which contribute to an effective immune response and, subsequently, a complete restoration of tissue function after resolution of inflammation [66]. In cancer, mechanisms that regulate gene expression in response to inflammatory stimuli link to its survival with the mechanisms that coordinate its phenotype and its function with the rest of the tissue [67]. This is often evident in severely compromised regulation of NF-κB activity, which allows cancer cells to express abnormal cohorts of NF-κB target genes [68]. The result is that not only the cancer cell functions abnormally but also the cells of surrounding tissue alter their function and cease to support the organism exclusively. Actually, research has been shown that several types of cells in the microenvironment of cancer may change their phenotypes to support cancer growth [69]. Inflammation, therefore, is a process that tests the verity of tissue components because the process requires coordination of gene expression between diverse cell types [70].

## 4. Therapeutic Approaches for Targeting NF-κB

Aberrant activation of NF-κB is frequently observed in many cancers. Moreover, suppression of NF-κB limits the proliferation of cancer cells. In addition, NF-κB is a key player in the inflammatory response. Hence, the method of inhibiting NF-κB signaling has potential therapeutic application in cancer and inflammatory diseases. Many natural products involved in anti-cancer and anti-inflammatory activity have been shown to inhibit NF-κB. Wedelolactone, an inhibitor of IκB kinase, suppressed both TNFα-induced IκB phosphorylation and NF-κB phosphorylation at Ser 536 and Ser 468 [71]. Parthenolide [72], and honokiol [73] also inhibits NF-κB pathway. Costunolide inhibited the activation of Akt and NF-κB and the expression of antiapoptotic factors B-cell lymphoma-extra large (Bcl-xL) and X-linked inhibitor of apoptosis protein (XIAP) in 11Z cells [74,75], magnolol inhibits ERK1/2 phosphorylation and NF-κB translocation [76], PI3K/Akt/caspase and Fas-L/NF-κB signaling pathways might account for the responses of A375-S2 cell death induced by evodiamine [77]. Oridonin [78], alantolactone [79], isoalantolactone [80], casticin [81], pseudolaric acid B [82], and jaceosidin [83], each of them has an inhibitory effect on NF-κB and its associated proteins. These compounds may inhibit one or more steps in NF-κB signaling pathway and its upstream growth factor receptors that activate the signaling cascade, translocation of NF-κB to the nucleus, DNA binding of the dimers, or interactions with the basal transcriptional machinery. In addition, many antioxidant compounds such as thiol antioxidants, calcium chelators, vitamin C and E derivatives, and alpha-lipoic acid have been used to inhibit hydrogen peroxide- or stimulus-induced NF-κB activation. Presumably, many of these agents act by scavenging reactive oxygen species (ROS) [84]. In addition, inhibitors of mitochondrial electron transport that suppress ROS production (like rotenone) or overexpression of antioxidizing enzymes, such as MnSOD and catalase, can block TNF-α-induced activation of NF-κB [85,86]. Caffeic acid phenethyl ester, a phenolic antioxidant and a structural relative of flavonoids, may directly interfere with DNA binding by NF-κB [87], and this effect on DNA binding was reversed by reducing agents like dithiothreitol [88].

Several nonsteroidal anti-inflammatory drugs (NSAIDs), such as aspirin, ibuprofen, sulindac and indomethacin, can inhibit activation of NF-κB in cell culture [89,90,91,92]. Actually, the majority of NSAIDs inhibit the cyclooxygenase enzymes (COX-1 and COX-2) at low doses [93]. The inhibition by aspirin is due to the irreversible acetylation of the COX site of prostaglandin endoperoxide synthase, leaving the peroxidase activity of the enzyme unaffected. In contrast to this unique irreversible action of aspirin, other NSAIDs such as ibuprofen or indomethacin produce reversible or irreversible COX inhibition by competing with the substrate, arachidonic acid, for the active site of the enzyme [94]. Thus, aspirin, unlike others, affects the COX-1 variant more than the COX-2 variant [95]. Low doses of aspirin are given immediately after a heart attack to reduce the risk of another heart attack or the death of heart tissue [96]. Aspirin is also effective at preventing certain types of cancer, particularly colorectal cancer and cardiovascular diseases at low doses [97,98]. The COX independent anti-inflammatory effects of NSAIDs include the inhibition of the cyclin-dependent kinase, Mitogen-Activated Protein (MAP) kinases and IkB kinase (IKK) that results in the inhibition of transcription dependent on NF-kB [99]. At higher concentrations, aspirin has also been shown to block NF-κB activity by directly binding to and inhibiting the kinase activity of IKKβ by reduction of binding ability to ATP [100]. In addition, aspirin has been reported to inhibit proteasome activity and consequently to interfere with degradation of IκB [101]. As such, high-dose aspirin therapy may have applications to diseases where NF-κB activity is involved, including cancer, diabetes and heart disease [102,103].

Glucocorticoids, such as dexametasone, prednisone and methylprednisolone, are used for their anti-inflammatory properties and to prevent allograft rejection through inhibition of NF-κB. Glucocorticoids inhibits NF-κB signal pathway through inhibition of DNA binding activity, and IKK activity and transactivation [104]. In addition, estrogen and certain selective estrogen receptor modulators (SERMs), such as raloxifene, can act through the estrogen receptor to inhibit NF-κB by a variety of mechanisms [105,106]. Several immunosuppressants target NF-κB. Several reports have shown that Cyclosporin A (CsA), inhibitor of B- and T-cell proliferation by blocking the activity of calcineurin, inhibits NF-κB induction [107]. Meyer *et al*. reported that CsA acts as a non-competitive inhibitor of the chymotrypsin-like activity of the proteasome, enabling it to block Lipopolysaccharide (LPS)-induced IκB degradation and p105 processing *in vivo* [108]. In addition, CsA prevents NF-κB nuclear translocation in stimulated T cells by preventing the inducible degradation of IκBα and IκBβ [109]. FK506 (aka tacrolimus) is an immunosuppressant that acts as a potent blocker of B- and T-cell proliferation. At least, in part, FK506, like CsA, acts by blocking the activity of calcineurin. However, unlike CsA, the inhibitory effect of tacrolimus on NF-κB appears, in some cases, to be specific for c-Rel, among the NF-κB family members. That is, FK506 can block c-Rel nuclear translocation (but not p50/RelA) after treatment of cells with phorbol esters and ionomycin [110].

Many human drugs that have been primarily characterized for activities other than anti-inflammatory or antitumor activity can also inhibit NF-κB. For example, Fibrates that is an inhibitor of PPARα [111], Gleevec that is an inhibitor of BCR-ABL [112], Raloxifene that is an inhibitor of Estrogen receptor [106], Rapamycin that is an inhibitor of FK-binding protein 12 [113], Triflusal that is an inhibitor of Cyclooxygenase-1 [114] and Troglitazone that is an inhibitor of PPARγ also inhibits NF-κB activity [115].

## 5. Conclusions

In conclusion, NF-κB controls expression of a number of genes that regulate immune responses, cell growth and proliferation, survival and apoptosis, stress responses and embryogenesis and development to a variety of stimuli. Aberrant NF-κB activation contributes to development of various autoimmune, inflammatory and malignant disorders including rheumatoid arthritis, atherosclerosis, inflammatory bowel diseases, multiple sclerosis and malignant tumors. Thus, inhibiting NF-κB signaling has potential therapeutic application in cancer and inflammatory diseases. This review provides the signaling mechanisms and biological functions of the NF-κB pathway, and the role of NF-κB in cancer and inflammatory diseases, and the multitude of NF-κB inhibitors that have been reported.
